# Understanding ‘passivity’ in digital health through imaginaries and experiences of coronavirus disease 2019 contact tracing apps

**DOI:** 10.1177/20539517221091138

**Published:** 2022-04-20

**Authors:** Alessia Costa, Richard Milne

**Affiliations:** 1 Wellcome Connecting Science, Engagement and Society, Cambridgeshire, Hinxton, UK; 2 Kavli Centre for Ethics, Science and the Public, Faculty of Education, University of Cambridge

**Keywords:** Passive data, digital health, surveillance, coronavirus disease 2019, contact tracing, ethics

## Abstract

Growing interest is being directed to the health applications of so-called ‘passive data’ collected through wearables and sensors without active input by users. High promises are attached to passive data and their potential to unlock new insights into health and illness, but as researchers and commentators have noted, this mode of data gathering also raises fundamental questions regarding the subject’s agency, autonomy and privacy. To explore how these tensions are negotiated in practice, we present and discuss findings from an interview study with 30 members of the public in the UK and Italy, which examined their views and experiences of the coronavirus disease 2019 contact tracing apps as a large-scale, high-impact example of digital health technology using passive data. We argue that, contrary to what the phrasing ‘passive data’ suggests, passivity is not a quality of specific modes of data collection but is contingent on the very practices that the technology is supposed to unobtrusively capture.

## Introduction

Visions of digital health set out by proponents and developers emphasise the potential to empower consumers by providing detailed, real-time access to data, and, ultimately shifting the balance of power in medical encounters; as in Eric Topol’s assertion that ‘the patient will see you now’ (Topol’s, 2015). Yet at the heart of this vision lie applications of so-called ‘passive’ data, that is, data generated by wearables, smartphones, and home sensors without the input of users (Cornet and Holden, 2018). Current and potential areas of application of passive data include diagnostics, self-management, and behavioural interventions targeting a range of conditions, most frequently mental health and physical activity but also, increasingly, chronic conditions and elderly care (Trifan et al., 2019).

The configuration of data as ‘active’ or ‘passive’ represents an important site of scientific but also ethical tension, at which developers and users negotiate the boundaries of acceptable and unacceptable forms of intrusive or intense monitoring. At the heart of these negotiations lie opposing views of the subject of digital health. On one hand, critical perspectives on ‘surveillance capitalism’ (Zuboff, 2018) and ethical and legal norms related to data collection have emphasised the importance of agentic engagement with data and the protection of autonomy in the face of business models based on the extraction of surplus value from data. In the context of digital health, the ethical value of *protecting* autonomy is extended to its *promotion* through narratives of ‘empowerment’ and users’ ‘activation’ (Prainsack, 2017).

On the other hand, the potential scientific, clinical and public health value of data from wearables, smartphones and home sensors, may emerge from a closer alignment with the methods, if not the values, of surveillance capitalist activities. This has implications for the role of users, and how to reconcile their position as passive producers of data points with their privacy and autonomy. This balancing is the subject of substantial normative ethical debate (Maher et al., 2019; Martinez-Martin et al., 2018). To date, however, there has been comparatively little attention to how these tensions are articulated and negotiated by users, not least because many of the proposed health applications of passive data are still at the phase of research and development and not available for public use.

To explore empirically how people imagine and live with passive health data, we examine public views and experiences of coronavirus disease 2019 (COVID-19) contact tracing apps in Italy and the UK. While the apps should be considered in the context of the specific challenges of the pandemic (particularly the infectious nature of the disease and the sense of global crisis), they can also be seen as the first significant introduction within national health care programmes of digital health solutions focussed on passive data. As such, they represent an important case study to analyse the roles ascribed to and adopted by the publics as active users or passive subjects of surveillance, and how these are negotiated and enacted within the socio-technical arrangements that passive data collection presupposes and reproduces. In tackling these questions, our aim is to deepen understanding of what constitutes ‘passive data’ and how ‘passivity’ is established and performed.

### Agency, passivity and the digital health subject

The development of digital health technologies is, in part, spurred by and predicated on the ideal of the active patient (Lupton, 2013). Commentaries on digital health highlight, and even celebrate, the potential to reposition patients/users from subjects of medical authority to autonomous individuals making informed choices about their own health (Erikainen et al., 2019). Comparatively less attention has been paid to “passive” data collection. Yet data produced without the active engagement of users form an important part of Big Data imaginaries – for Ruppert, for example, “the production of big data imagines subjects as *passive actants* where technologies are one-way tools for extracting data about them” (Ruppert, 2018: 21, emphasis added).

In the context of health, configuring users as passive actants is key for researchers and developers to realise the potential value of data for clinical and scientific research. As one researcher from a leading company in the field remarks, passive data offer two advantages compared to other types of data:

First, they are unobtrusive placing no burden on the subject beyond the normal use of a smartphone. Second, they are ecological since the smartphone data is captured in the natural environment (Dagum, 2018: 2).

The *unobtrusiveness* and low ‘burden’ of passive data make them amenable to being gathered pervasively and continuously, enabling the production of ‘denser’ assessments of targeted functions in ‘nature’. Moreover, the capacity to capture data that exude from daily activities can significantly expand the scope of what is considered health-related data. While self-tracking technologies usually focus on data that have a clear relationship with health and wellbeing (e.g. symptoms, physiological values, sports performance), the types of ‘passive data’ being used or considered for health-related purposes encompass a much broader range of bodily functions and embodied practices, including eye movements (Bott et al., 2017), hand movements (Tarnanas et al., 2015) and swiping patterns on mobile phone screens (Dagum, 2018).

On the other hand, commentators and scholars (including Dagum, Martinez-Martin et al. 2018) have pointed out that such data practices raise questions regarding privacy and meaningful consent, data minimisation and secondary use, as well as transparency and accountability (Maher et al., 2019; Martinez-Martin et al., 2018). As with wider critiques of surveillance capitalism (Zuboff 2018), *passive data gathering* risks rendering users into *passive subjects* whose autonomy and capacity for self-determination are eroded. From this perspective, passive data both exemplify and exacerbate tensions between empowerment and surveillance, autonomy and control, that lie at the very heart of contemporary data practices (Darmody and Zwick, 2020).

Central to these debates is the question of the subject’s agency, and how it is configured vis-a-vis digital technologies. Sharon (2017) and Lupton (2012), for example, point out that, while emphasising empowerment, self-tracking is a “paradigmatic practice of contemporary surveillance society” (2017: 98), contributing to the disciplining and monitoring of populations through the extension and incorporation of the clinical gaze (see also Erikainen et al., 2019). Schüll (2016) and Lindner (2020) further problematise these questions through their analysis of the digital nudge. While promising to empower users to take active charge of their life, they note, digital health technologies shift the effort to the tools to assess information, calculate risks and nudge users to act in responsible ways (Lindner, 2020). The technology, Schüll argues, provides discernment and control precisely where these are lacking, thus positioning the user as a “*passive*, choosing self” (2016: 14). Till, therefore, asks whether the user of self-tracking technologies is best conceived of as active or ‘automated’ Till (2019). Similarly, Fors and co-authors write:

Such technologies thus allow only for certain programmed futures by deciding in advanced what count as usable data, how these data should be interpreted and visualised and how people should engage with these data (2019: 94).

Importantly, scholars adopting a socio-material approach have examined how users’ roles are articulated in practice in ways that defy and creatively appropriate the often-narrow agenda of digital health tools (Nafus and Sherman, 2014; Sharon and Zandbergen, 2016; Sharon, 2017). This literature has expanded the analytical focus from the question of how users rationally scrutinise data to how they gather, curate, review and incorporate them in everyday life (Kristensen and Ruckenstein, 2018; Lupton, 2017; Lupton, 2018; Pantzar and Ruckenstein, 2015; Schüll, 2016; Weiner et al., 2020). A key contribution of this literature has been to direct attention to the mutual processes through which ‘people make data and data make people’ (Lupton, 2018), thus reframing agency as distributed. These theoretical perspectives conceptualise user roles as shifting and mediated by the socio-technical arrangements that specific modes of data collection and processing require and reproduce. Specifically, they bring into focus the sensorial and material dimensions of data practices, raising questions as to how users’ position might be reconfigured when practical engagement with data is automated and made ‘passive’.

### Passive data collection and the pandemic

From the earliest stages of the COVID-19 pandemic, researchers and developers in the public and private sector have looked at ways to take advantage of the potential of data generated through everyday activities. FitBit, for example, launched a study suggesting that users’ heart rate data might usefully identify early signs of the body’s response to the virus (Heneghan, 2020). Others have investigated the potential of wearable sensors for temperature (Smarr et al., 2020), voice analysis (Brown et al., 2020), or combinations of heart rate, sleep and activity data collected by digital devices (Quer et al., 2021). At a policy level, proprietary data collected by Google and Apple’s location services were used by the UK government to track communities’ responses to lockdown (UK Government Cabinet Office, 2020), while Google Health Trends data on COVID-related search terms formed part of Public Health England’s early community surveillance platform (Public Health England, 2020).

It is through COVID contact tracing apps, however, that passive data have been most visible in public imagination and debate. From the beginning of the pandemic, a number of countries explored options to take advantage of the promised speed and scalability of digital contact tracing, often based on passive data (Ada Lovelace Institute, 2020). The governments of countries from South Korea to Vietnam, Taiwan and Israel deployed a range of data surveillance techniques, using data from telecommunication networks, GPS, credit card transactions and social media to trace contacts that people might be unwilling or unable to report (Lewis, 2020). The most widespread digital contact tracing tool has been dedicated apps that monitor proximity and alert users if they have been exposed to the potential infection through extended contact with an individual who subsequently has a positive test for coronavirus. These have primarily drawn on an application programme interface (API) framework developed by Google and Apple, the owners of the two main mobile operating systems, Android and iOS.

Both England and Italy, the two countries that form the focus of this study, released contact tracing apps that use the Google and Apple API framework. In Italy, the *Immuni* app was launched in June 2020. After an initially slow adoption, the number of downloads significantly increased over autumn, concurrent with a resurgence of infections. As of January 2022, the app has about 19 million downloads from an overall population of about 60 million people, although the number of notifications sent remains relatively low (just under 150,000 from about 48,000 ‘positive users’) (Immuni, n.d.). After several delays, the National Health Service (NHS) COVID-19 app for England and Wales (Scotland and Northern Ireland use a different app) was launched in September 2020. As of January 2022, it has been downloaded 26 million times, from an overall population of about 60 million people. It sent more than 10 million alerts to users, registering a significant increase following the easing of most COVID restrictions in the summer of 2021 (NHS, n.d.). On the other hand, numbers show a steady decline in the use of the more ‘active’ features of the app, such as the ‘QR check in’ that allows users to report their location when entering restaurants, shops and public venues, so that they can receive alerts if they visited a COVID hotspot (NHS, n.d.).

The development of the apps attracted significant public and ethical scrutiny, particularly with regard to issues of privacy, security, and, to a lesser extent, access and equity (Gasser et al., 2020; Kind, 2020; Morley et al., 2020; Parker et al., 2020). The novelty of the tools, the intensity of debate and the need for high uptake rates, meant that much attention was directed towards addressing possible concerns around privacy. Other questions, however, have received far less scrutiny (Morley et al., 2020; Sharon, 2020). In an early commentary, Hoffman and colleagues focussed on how app design aims to create a ‘seamless’ experience that relies on users doing nothing apart from installing the tool on their phones and enabling continuous data collection (Hoffman et al., 2020). Such seamlessness, Hoffman and co-autors argue, positions users as an “unreliable herd in need of monitoring” (2020: 111), rather than engaging them as active data subjects, for example through asking them to utilise features like the QR check in. As the authors point out, the use of passive data in this context is potentially at odds with the notion of autonomous, responsible citizens which informed the governance and design of voluntary apps. Moreover, they suggest, the very use of apps might lead users to delegate responsibility to the tool, thus producing a more passive engagement with risk or even unsafe behaviours.

In qualitative work with users of the Chinese contact-tracing and quarantine app Health Code – among the first to be implemented – Liu and Graham (2021) describe the range of positions their participants took, emphasising how these are neither predetermined nor fixed, but evolve and may be contradictory. This is because people perceived the app through context-specific sensemaking practices, meaning they engaged with it through past experiences, daily interactions and future anticipations. In their interview study on public perceptions of the contact tracing apps in German-speaking countries, Zimmermann et al. (2021) similarly show that participants linked the tools to their previous engagement with digital technologies, particularly other familiar applications that collect data. This framing of the technology gave rise to scepticism and distrust for some, through the association with surveillance, but also contributed to normalising the novelty of digital contact tracing in the eyes of others.

### Methods and materials

We draw on data from repeated interviews with older adults in the UK and Italy and documentary evidence on the respective apps in the two countries. The countries were selected opportunistically to widen the scope of the research to non-English-speaking contexts.^1^ Despite significant differences in the following stages of the pandemic,^2^ at the time of the study, the two countries saw similar rates of infections and had adopted similar public health restrictions, with the main difference being the mandatory use of self-declaration forms for any travel outside one’s home in Italy during the first lockdown. As mentioned, both countries released apps that used the Google and Apple API framework following the easing of initial restrictions.

Documentary evidence on the apps was gathered from public-facing documents, including governments’ campaigns, media reports, published literature, and the websites of the Google and Apple API framework. Further data on the individual apps were gathered through established methods to systematically explore and document the various stages of an app’s download, activation and discontinuation (Light et al., 2018). The interviews were conducted as part of an on-going study on digital health technologies. As we were finalising plans for interviews with older members of the public to explore their use of digital technologies, the pandemic struck and national restrictions were introduced.

Despite the disruptions to our initial plans, and the challenges of conducting research remotely, the pandemic provided a methodologically productive time to explore questions related to digital health, as many of us became more reliant on digital technologies to keep up daily activities during the lockdown, and the launch of the COVID apps fostered public debate on digital health data. The pandemic also raised significant challenges for research, as it meant conducting interviews on a potentially sensitive topic (i.e. health risk) at a time when participants might be experiencing significant stress around their own health and that of their loved ones, as well as being affected by the disruption, isolation and uncertainty caused by the on-going crisis. We, therefore, decided to adopt a longitudinal approach and conducted three interviews at a few-weeks distance from each other. This allowed us to build rapport with participants and approach the topic gradually, leaving space to identify any needs for support. The approach also enabled us to explore emerging and changing imaginaries and practices of digital health, discussing broader changes due to the pandemic and contextualising participants’ views and use of digital technologies in their daily lives. The revised study protocol was ethically reviewed and approved by the Wellcome Genome Campus Research Governance Office.

Using a semi-structured schedule, the three interviews explored: (a) people’s everyday lives and use of technology prior to and during the pandemic; (b) initial views and experience of the COVID apps which were about to be/had just been launched; (c) any changes in participants’ experiences of the COVID apps and their views on digital health technologies more widely, specifically in relation to other diseases associated with ageing. The study took place between April and October 2020, covering the last weeks of the first period of ‘lockdown’, a time of relative easing of COVID restrictions over the summer, and the beginning of the so-called ‘second wave’ of infections in autumn. The first interviews were conducted in April and May (UK) and June (Italy)^3^, the second around two weeks later and the third between September and October 2020. Here we focus primarily on data from the second interviews (unless otherwise indicated), as more directly focussed on the contact tracing apps.

In total, 30 participants (UK = 19; IT = 11) aged between 50 and 88 years took part. They included 19 women (IT = 8, UK 11) and 11 men (IT = 3, UK 8). The majority of Italian participants were from the northern regions, including areas among the first to be affected by the pandemic. Roughly half resided in rural areas and half in urban centres. UK participants were based in the East and South East of England, 14 in rural areas, 5 in urban areas. Age (i.e. being over 50)^4^ was the only criteria for inclusion. Participants were recruited through social networks, local print media, social media and snowball sampling until the established quota was reached. Participants received information about the study over the phone, were offered a written information sheet and contacted again to confirm interest. All were interviewed three times, and verbal consent was recorded before each interview.

Participants included those who did, or planned to, use the app (*n* = 15 in UK; 5 in Italy) and those who did not. Among the latter, three had intended to download the tool but were unable to due to a lack of access to technology, while the remaining would not get the app for personal preferences (*n* = 1 in the UK; 6 in Italy). Participants had different degrees of familiarity with digital technologies, and while all owned a smartphone, not all these smartphones were either new enough or sophisticated enough to act as digital contact tracing tools.

### Configuring the active user

As Hoffman notes, from the first phases of the pandemic, passive data became the go-to solution for digital contact tracing (2020). The minimal input required from users was “cast as a virtue through a conceptual coupling with lowered cognitive load, and thus freedom” (Inman and Ribes, 2019: cited in Hoffman et al., 2020: 108). Furthermore, technologies for passive monitoring like Bluetooth and GPS made it possible to generate ‘dense’ data on proximity, length of exposure and number of contacts, including those that the subject might be unaware of or unable to identify. As noted, these justifications draw on wider, existing imaginaries of passive data. Thus, the question was not so much *whether* to use passive data, but *what configurations* of passive data might best ensure effectiveness as well as public trust and uptake.

Developers of the two apps considered here opted for Bluetooth over GPS, as this allowed recording proximity and contacts without ‘tracking’ users’ location. A key issue was the choice between ‘centralised’ and ‘decentralised’ systems. In the first, data are stored and processed on a central server, in the latter this happens on users’ devices and data are only shared when users log a positive test result or are notified about at-risk contacts. In public and media debate, the decentralised option was often depicted as more privacy-focussed, in what some have considered a one-sided and myopic representation (White and Van Basshuysen, 2021). Google and Apple joint API framework, adopted by both apps in question, famously favoured this ‘privacy preserving’ approach. The UK initially pursued a different strategy, seeking to develop an in-house, centralised app by NHS England’s digital team. The plan, however, was eventually abandoned because the technical limits imposed by mobile phone developers did not allow for ‘passive’ data gathering, meaning Bluetooth contact tracing automatically stopped working if users did not engage with the apps for a certain period time (Department of Health and Social Care, 2020).

**Figure 1:**
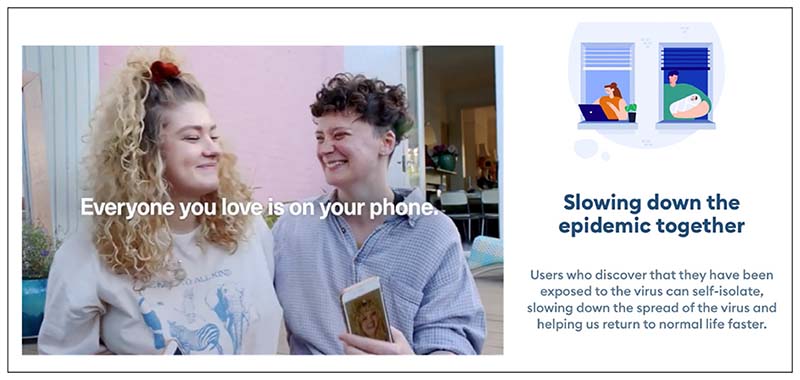
On the left, an image from the UK government public campaign (NHS coronavirus disease 2019 [COVID-19] App, 2020). On the right, a page from the Italian Immuni app.

Amid public concerns over privacy, public messaging around the apps emphasised the active role of users. Government campaigns, official websites and the content and tone of the apps positioned users as responsible and solidaristic citizens whose choices could help to protect those around them and society at large (Figure. 1). The UK government campaign to launch the app, for example, featured individuals, couples and families showing pictures of their loved ones on their phones accompanying the message:

Everyone you love is on your phones, now so is the app that helps protect them from Coronavirus (NHS COVID-19 App, 2020).

Building on the role of mobile phones in keeping people connected and maintaining social relationships under conditions of isolation, the campaign highlighted the social value of the app as a way to take care of others. These imaginaries resonate with messages that emphasised the apps’ effectiveness, describing it as “the fastest way” to be notified of possible risks and “protect your loved ones and community”. Drawing on similar imaginaries, the Italian app was also presented as a simple yet powerful tool to protect oneself, one’s family and country (*Proteggi te stesso, la tua famiglia e il tuo Paese*) (Immuni, n.d.).

A key feature of these discourses is the way in which they mobilise sentiments of solidarity, civic duty and social responsibility that characterised official and informal responses to the pandemic, particularly in the early phases of the crisis, when the outpouring of applications to become NHS volunteers, the creation of mutual help groups and the scenes of people cheering up each other from balconies caught the popular imagination as examples of community values at a time of crisis. It was these values that the Italian Health Minister appealed to, on the eve of the first European lockdown in March 2020, when he urged citizens to play their part because “there’s no magic wand, only the individual effort of the collective” (RaiPlay, 2020, March 8).^5^

As everyday mundane actions (Who have you met? What have you touched?) were brought into new focus, health risks were framed as a relationship between the individual and collective, where the wellbeing of the latter became the responsibility of the former (Petersen and Lupton, 1996). Reinforcing familiar discourses that informed the public response to previous pandemics (Fee and Krieger, 1993) and pervade the current rhetoric surrounding digital health (Erikainen et al., 2019), we have all been called upon to ‘stay alert’ and ‘act responsibly’ in order to protect ourselves and others (Kieslich et al., 2020). Maybe more clearly than ever before, these calls to individual responsibility were overtly framed in terms of alleviating the burden on shared health care resources and protecting the most vulnerable, as expressed in the UK government slogan at the time of the first lockdown: “Stay home. Protect the NHS. Save lives”. In this way, the pandemic made apparent the biopolitical dimensions of self-discipline and self-monitoring practices, including digitally mediated ones (Lupton, 2020).

This relation between self-monitoring practices and collective wellbeing has been central to the apps’ vision, as in the campaign ‘The more we are, the better we are’ (‘*Più siamo e meglio stiamo*’), launched by the Italian government to encourage adoption of the app and “promote a sense of personal responsibility and belonging to the national collective” (Presidenza del Consiglio dei Ministri, 2020). Official communication on the apps has consistently avoided the imperative tone deployed for other COVID measures (e.g. ‘stay home’), placing emphasis instead on the voluntary and solidaristic contribution of individual users (Prainsack, 2020). The dynamics of infection control have further reinforced this dimension of imagined user experience. At a time when responsible behaviour became synonymous with lack of action and isolation, downloading the app was presented as a way to reclaim one’s role as an active and responsible member of the community (cf. Presterudstuen, 2020).

Users were thus framed as autonomous individuals who endorse and freely choose to participate in data practices designed to ease flows of passively collected data. When downloading the app, users were asked to explicitly consent to proximity detection and exposure notifications. The ‘onboarding’ process, through which users download and install the tools, was designed to be intuitive, seamless and quick, to minimise burden and maximise uptake. Text was kept minimal and friendly, while lengthier and more detailed information on terms and conditions, data policy and privacy was conveniently provided in separate pop-up pages. Information on privacy occupied an important space in both apps and was presented early on in the onboarding process, signalling the perceived importance of the issue. Users were therefore reassured that the apps had no way to know their identity, contacts and movements, and their data would remain anonymous.

**Figure 2:**
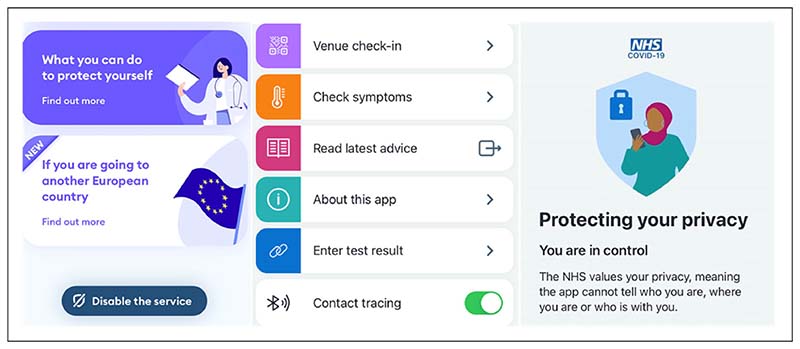
A section of the main pages of the apps Immuni (left) and NHS coronavirus disease 2019 [COVID-19] (centre and left), including the toggle to disable contact tracing.

Both apps stipulated that in case users tested positive, they could choose whether or not to allow use of the data stored on their devices. The choice was voluntary and framed through the participatory rhetoric of ‘data sharing’. Moreover, both apps provided the option to disable proximity detection at any time while still using other functions such as symptom checking and the QR check in (Figure 2). The NHS app was particularly interesting in these regards, as the choice of the ‘toggle’ tool to switch contact tracing on and off appeared to deliberately emphasise that consent was not final and users retained control over when and where they accepted passive monitoring.

Users’ experience was thus carefully designed to emphasise autonomous choice, proactive engagement and solidarity. Their engagement with the tools was construed to position them as active subjects, while also facilitating unobtrusive, burden-less data collection. In doing so, the apps’ governance was aligned with ethical norms protecting autonomy and privacy, even as the need for conscious, agentic interaction was obviated by design.

### Taking action, becoming passive

Participants who had downloaded the app, or said they would, justified their decision by comparing it to other ways they were *acting* to manage the risk of infection, such as respecting social distance, wearing face coverings and paying attention to hands hygiene. These activities had a strong solidaristic connotation, as people recognised that by minimising risks for themselves they were also contributing to protect others (Galasso, 2020; Kieslich et al., 2020). This was particularly true in the case of the app, whose value was primarily to improve contact tracing rather than avoid immediate risk for the user. For some participants, therefore, the decision to download the app was part of a broader idea of pandemic citizenship, with some explicitly couching their choice in terms of “civic duty” (IT9, UK5) and “social responsibility” (IT7, UK5). An Italian participant thus commented:

As a member of society, what I can personally do to protect the health and wellbeing of others is having a tool which can help public health, and help other people to protect their health. My own health too, of course, but it’s not that Immuni…it tells you that you’ve been in contact [with someone who tested positive]. Then it’s on me to take precautions, but in doing so I also take them for others. So, I download it to make sure I act as a good citizen really (IT11).

As a UK participant described, such solidaristic concerns may even involve the acceptance of some cost to individual privacy:

I can see that other people, yeah, you know, they can say it’s sort of Big Brother watching you and tracking you the whole time. I think if it’s for the common good, it’s okay (UK5).

The participant commented that the social value of the app provided a strong justification for the potential exposure of her personal life, pre-empting concerns about privacy or the uncertain benefits for herself and her family:

I think if an app was released, I think that you would feel a social responsibility to use it on your phone, even if you were suspicious of, you know, data harvesting and the efficiency of the app, the ability of the app to help with the pandemic. You’d certainly feel bad if you didn’t, certainly feel guilty if you weren’t using it (UK5).

As she described, solidarity can be closely associated with duty and responsibility – and the perceived consequences of failing to make oneself available for such surveillance. These discourses resonate with the imagined role ascribed to users as active participants in the collective effort at containing the pandemic, as articulated in official communication about the apps.

At the same time, however, interviewees framed the apps in the context of prior experiences of digital technology and surveillance, emphasising their lack of control over, and awareness of, how data about them were generated and used. Almost all participants mentioned direct, personal experiences of surveillance, most commonly in the case of targeted advertising. They articulated a variety of views on surveillance, as both innocuous and “spooky” (UK3), habitual and uncanny, and many voiced disquiet about the perceived lack of privacy. Invariably, all described surveillance as ubiquitous and unavoidable, as in the following excerpt:

We are always localised (localizzati), even the tablet we leave at home is localised. So, I don’t see anything new about this. It doesn’t mean that they look at us … But if you get a message asking you to review your local grocery store, what does that mean? That someone knows I’ve just been there, right? It’s the same thing with this app (IT10).

Participants often referred to the idea of control to articulate these experiences, but the concept was mostly used one-way. Participants seldom commented on the technical aspects of the apps design, and how it allowed or restricted users’ control on their data. Instead they often described how the app exposed them to someone else’s control. This was particularly true for the Italian interviewees:

It immediately gave me the impression of an exaggerated control (controllo), which could then serve other purposes, unrelated to health (IT4).

Even when participants appeared unconcerned about the possible implications of surveillance, downloading the apps was seen as relinquishing control over what data could be gathered about them, by whom and for what purposes:

I think the worry that most people have is that people are taking your personal information. And I think they probably are. But I think they are in most things when they’re online, actually (UK13).

These views were mediated by previous experiences of digital technologies, and how these could “leave yourself open” (UK19) and exposed. In participants’ experiences, flows of digital data remained obscured, but their effects (e.g. targeted advertising) were clear and visible. This created a sense of imbalance between what the interviewees felt they knew and controlled about their data, and what others, often a generic ‘They’, could learn and do. Data were *about* users but gathered, viewed and used by others. ‘They’ could “take”, “collect”, “steal” (*trafugare, rubare*), “leak”, “amass”, even “lose” or “mess up with” (*scambiare*) data. Participants, on the other hand, hardly figured in the position of acting subjects in relation to their data. When they exercised agency, it was to “give”, “release” (*rilasciare*), “communicate” (*comunicare*) or “spread” (*diffondere*) data, but hardly to control them. These passive data practices were rarely couched in terms of “sharing”, as this would imply retaining some sort of relationship with the data. Instead, participants’ responses suggested that at the very moment when data were captured by the devices they ceased to be under users’ control:

There’s no way back; unless you throw away your smartphone, then no one can touch you anymore. But even then, your data is already gone (IT9).

Against this background, a number of participants expressed support, at least in principle, for mandatory enforcement of the apps or even for more intrusive forms of surveillance, such as monitoring phones instead of promoting the use of voluntary apps. One participant for example explained:

We’re controlled anyway, we might as well force people to be more controlled for the time being. If they want they control us (IT3).

Interestingly, participants who decided not to download the apps also shared these views, particularly among Italian interviewees. Those in favour of these hypothetical measures invoked the need for large-scale uptake to ensure the effectiveness of digital contact tracing, stressing that individual privacy and autonomy should be balanced against the public health benefits of digital surveillance. Elaborating on this point, an interviewee explained:

I would have preferred that, if it was really so important and necessary, that it had come from the government so that you don‘t have to download anything and it‘s mandatory. Once they had checked its validity and, how can I say, ensured all protections were in place, I would have used the iron fist so to speak. I would have made it mandatory, so that it applied to everyone (IT1).

In this case, accepting the possibility of being subject to external control was framed as a form of responsible action, not because it followed from autonomous choice, but because it presupposed participation to a collective whose norms and interests preceded the individual’s decision to contribute to them (Kelty, 2020).

### Living with passive data

Overall, participants recognised the value of passive monitoring to help detect health risks. Discussing the imminent release of the app in the context of the easing of COVID restrictions, a UK interviewee commented that the tool would make her feel safer, especially in light of her lack of confidence in others’ behaviour. She explained:

Because this is such an uncertain situation and desperate though I am for life to go back to normal, I do want a degree of control over what happens. And, I think that this app would be a way to kind of ease us out of lockdown without this Corona-phobia taking over (UK2).

In this case, outsourcing the management of risk to the app was seen as enhancing the user’s capacity to act in her everyday life.

Other participants, however, were less persuaded that the app would have a significant impact on their lives. In fact, some felt that it was not doing much at all, to the point that it was almost like not having it:

So, my daughter told me it’s a good thing to do and she downloaded it in secret on my phone. But so far, it’s like not having it, it never rings. It’s like I don‘t even have it. But, in any case, I’ve got it downloaded (IT7, third interview).

As the quote highlights, the app afforded limited scope for active engagement (“it never rings”). Participants did not report receiving notifications or using any of the more active features of the app. Equally, they did not describe changing their behaviour significantly because of the extra protection supposedly afforded by the tool. For most, the role of the app remained rather marginal:

I never received any notifications, so once in a while I open it, just to check it didn’t get deactivated. But nothing, it’s working but I haven’t received any notification (IT11, third interview).

These responses contrast with the view of the app as enhancing users’ agency, as well as with the narratives of social responsibility discussed earlier. They paint a picture of a much more passive user whose everyday behaviour is unaffected by the tool and whose input is limited to checking the app has not stopped working. If the choice to download the apps was characterised by high expectations and strong concerns, their role in people’s everyday life appeared in contrast rather mundane, to the point users almost forgot about them.

Contrasting views about the value of the apps, as either enhancing users’ agency or doing nothing, were not mutually exclusive. In fact, participants often highlighted how the role of the app in their lives could vary depending on the very practices and spaces that the technology was supposed to passively monitor. A number of interviewees for example commented that the app would be of limited value in familiar situations:

I don’t travel outside of the village, so I don’t … I might as well download it whilst I’m here, just spending my afternoons in retirement. But for the moment, I didn’t see the need. I thought, if I had to take a train, a plane or bus, but if it’s just me and my car, or my car and I […]And if there ever is anyone else in the car with me it’s my niece, so I haven’t … (IT10).

On the other hand, the app could become quite helpful if visiting unfamiliar or crowded places:

Everybody in this village, pretty much knows anybody who’s had it…Everybody’s pretty much been locked down. So, I feel that [I can rely on] my own risk assessment, that they’re relatively safe people to stay around as long as you’re socially distanced. Where it would probably be quite useful is, in a couple of weeks’ time I need to go to [nearby town] to visit IKEA … In all of those places, the app would be quite useful, not necessarily to protect me at this moment, but to give me information afterwards. If it was really working properly, then, you know, it would allow me … Yeah, I would get some kind of alert saying that you were near someone yesterday who’s tested positive (UK8).

The excerpts highlight the contrast between the spatial dimension of risk as captured by the apps, in terms of physical proximity, and as assessed by potential users. In the latter case, proximity assumes a sensorial and relational meaning, which is not simply about physical distance but about social relationships (e.g. “everybody knows anybody”, “my niece”) and spatially situated practices (e.g. the village vs. the outside). In situations that participants could not decipher on the basis of their senses and judgment alone, passive data could come alive; it materialised otherwise undetectable risks and enabled participants to act meaningfully. On the other hand, where participants felt they had control over the situation, either because the possibilities to come in contact with strangers were limited or because they were confident they would know if someone they met later tested positive, the app faded into the background and stopped being relevant.

A number of participants commented that the lack of active engagement with data contributed to the perceived irrelevance of the app. Participants, for example, described how exposure notifications might be difficult to decipher if one had no way to interrogate data about the specific contact:

Knowing that without realising it you have come in contact with someone positive might alarm you: Have I been far enough? Did I keep the distance? Did I run any risks? (IT1).

The time lag between exposure and notification could add to the challenge of making sense of the data-driven instructions, as participants might struggle to reconstruct the circumstances of the encounter. One participant therefore noted that carrying the app would make her feel “more paranoid” (UK5).

The inscrutability of the process of data collection and analysis meant that users had limited insight into the type of the risk involved. For one participant, this made her feel at the mercy of the tool, which she perceived as highly sensitive but also dangerously non-specific. She feared that the app could notify her even in case of brief contacts that posed no actual risk, leading to almost complete paralysis:

Just think how many times you can encounter someone who then tests positive. Maybe you’ve just completed a period of self-isolation and I leave the house and I encounter someone. […] You see? You’d have to stay at home all the time! (IT7)

In her view, outsourcing control to the app could impede rather than enable action. A similar situation, in fact, happened in England in the summer of 2021, when the easing of COVID restrictions at a time of still relatively high rates of infections led to a sharp increase in the number of people who were instructed to self-isolate by the app. The so-called ‘pingemic’ resulted in disruptions and shortages of key workers, eventually leading to the decision to alter the app parameters to trigger notifications (Rimmer, 2021).

A related issue highlighted by participants was the actionability of the information provided by the app. One interviewee for example commented:

I think the problem that we’ve got, certainly in my industry is that people are trying to work as much as possible. And if the restrictions get any greater and there‘s no financial support they will be tempted to sort of go under the radar and carry on and do as much as they can, and I think they will be wary of using an app which tracks their movements (UK10, 3).

Similarly, an Italian participant discussed her concerns about the news that users had to wait longer than required to end the self-isolation period due to reduced testing capacity. In these cases, participants commented that not exposing themselves through the app was essential to retain the ability to act. Significantly, this was seen as compatible with the role of responsible citizens, as participants stressed the importance of taking matters in their own hands (by wearing masks and respecting social distance) instead of entrusting the app to materialise risks that they lacked the capacity to act upon.

In light of these issues, a number of participants, particularly among the Italian interviewees, reasoned that paying attention to everyday behaviour was more important than having the app, and relying on the latter could imply exposing oneself to the very risk the tool was aimed to contain. These responses emphasised people’s proactive role in managing risk, as described by the following participant:

I don’t move much, if anything I travel to the nearest town but since when this thing started I’ve been there maybe three times. When we’ve been there [for my wife’s hospital visit], my wife’s gone in but I waited outside in the parking lot. I don’t go close to people. I don’t smoke, don’t drink, I’ve quit smoking and if I have to go somewhere I take my own bottle [of water] with me and I make do with what I have (IT2).

## Discussion

Growing interest and investment is been directed towards health-related uses of passively collected data, with critics raising concerns over threats to users’ privacy and proponents envisioning a future where pervasive, continuous monitoring will lead to ever more precise and effective insights into one’s health and wellbeing. These positions can reproduce polarised views of digital health technologies as either tools for individual and collective good or forces of ever-expanding surveillance (Sharon, 2017). More broadly, debates on passive data both amplify and exacerbate tensions between the incentive to widening the production, sharing and harnessing of data and the challenges of ensuring consent, control and autonomy of individual data subjects. Empirical studies on the social and ethical implications of passive health data, however, remain scarce.

In this article, we have explored how questions related to passive data are negotiated in practice by individuals who are target users of such technologies, focussing in particular on the relations between passive data and user agency in everyday life. We have done so through the example of the COVID-19 contact tracing apps. While people’s experiences of the apps should be considered in view of the circumstances of the COVID pandemic, they also provide an important case study to explore users’ engagements with passive data in practice. Moreover, the prominence of the apps in the public discourse and their role in the public health response to the pandemic have expanded the pool of potential users to include groups, such as older people or people less digitally included, who have been less well represented in predominant accounts of digital health focussed on ‘self-trackers’ and ‘quantified selfers’. As such, people’s experiences with the apps have wider relevance to understanding the implications of passive data beyond COVID.

Participants’ experiences of the contact tracing apps resonate with existing critical studies that emphasise the potential of passive data to leave subjects unwillingly or unwittingly exposed to surveillance (cf. Zuboff, 2018). However, in the specific context of the pandemic, becoming passive subjects of surveillance was not only an (unintended) outcome of data practices, but could be actively accomplished, performed and valued. In line with the findings of other emerging studies (Mouter et al., 2021; Williams et al., 2021), the societal value of the apps and their place in responsible pandemic citizenship was an important reason to download the tools, acknowledged even by those interviewees who decided not to take part in digital contact tracing. As private acts of movement and encounter became objects of public health value, becoming the subject of surveillance emerged as the product of both institutional configurations, which gave particular meanings to surveillance and produced incentives to expose, and the actions of subjects who personally endorsed and opted in to such exposure, or at the very least tolerated it (cf. Ball, 2009).

This has consequences for the ways in which the implications of passive data are conceptualised and addressed. The existing literature within and without digital health tends to conflate questions of agency with individual autonomy, focussing on privacy and control as coded into the design of data infrastructure and technologies (e.g. swipe left or right to enable data sharing). Our analysis, however, suggests that the subject’s agency is also dependent on the wider societal arrangements that give meaning to data flows (Ball, 2009; Cohen, 2012: 107 − 152). Participants’ accounts of living with passive data emphasised how the role data played in their lives was contingent on the very practices that the technology was supposed to passively capture. The app could fade into the background of people’s lives, becoming irrelevant in familiar circumstances, or even a demonstration of *irresponsibility* when contrasted with a more ‘active’ approach to managing risk. Conversely, it could be salient and meaningful where people were not able to have full control of the situations in which they found themselves, becoming an unobtrusive part of ‘active’ engagement with COVID risk.

Participants also highlighted the (lack of) engagement with data, particularly the limited feedback the app provided apart from exposure notifications. Unlike self-tracking tools, COVID apps did not afford users the capacity to visualise, interpret and derive insights from their data, or to use data-driven instructions to navigate situations in real time. It was this limited feedback and agential engagement, and not simply the lack of input at the point of collection, that made data ‘passive’ in the sense that they produced no tangible effects in users’ daily encounter with risk. Depending on the social and institutional context in which users found themselves, ‘outsourcing’ control to the app to manage their potential exposure *to* infection and their exposure *of* information about themselves acted as either a way to retain and enhance agency, or failed to afford users the capacity to act differently.

In conclusion, our data show that the nature of, and users’ relationship with, passive data were never fixed. Contrary to what the term ‘passive data’ suggests, passivity is never a quality of specific modes of data gathering alone. In fact, the distinction between active and passive data can be difficult to define or observe, and the boundaries between the two are constantly situated and re-enacted in practice. Passive data are thus only such in relation to their subjects and to the contexts in which they are generated and circulated.

## Limitations

The countries were not chosen for particular differences in apps configuration, public response to the pandemic and/or cultures of privacy. Participants’ socio-demographic details are not representative of the wider population. These were not major limitations for this study, as we focussed on the conceptualisation of passivity. Future research however might examine more closely the impact of cultural and socio-demographic differences on users’ experiences of passive data.

## Footnotes

1 For an analysis of views on COVID apps in different European countries see Lucivero et al. 2021.

2 With the progress of the respective vaccination campaigns, public responses to the pandemic in the two countries started to diverge quite radically, especially with regards to immunity passport (mandatory in Italy to access public transport, private and public venues and workplaces, and only required in the UK for large events with several thousand people) and vaccine mandates (Italy became one of the first countries to make vaccines mandatory for all citizens above the age of 50).

3 The different start date was due to the longer time to obtain ethical approval for research outside of the UK.

4 This is because the wider study focused on digital health technologies for older adults.

5 See also Raffaetà (2020).
